# Clinical Efficacy of Multimodal Exercise Telerehabilitation Based on AI for Chronic Nonspecific Low Back Pain: Randomized Controlled Trial

**DOI:** 10.2196/56176

**Published:** 2025-05-22

**Authors:** Chongwu Xiao, Yijin Zhao, Gege Li, Zhuodong Zhang, Siyu Liu, Weichao Fan, Jinjing Hu, Qiuru Yao, Chengduan Yang, Jihua Zou, Qing Zeng, Guozhi Huang

**Affiliations:** 1 Center for Rehabilitation Medicine, Zhujiang Hospital, Southern Medical University, No.253, Industrial Avenue Middle Guangzhou, Guangzhou, 510280, China, 86 19543576136; 2 School of Rehabilitation Sciences, Southern Medical University, Guangzhou, China; 3 Department of Rehabilitation Medicine, The Second Affiliated Hospital of Guangxi Medical University, Guangxi Medical University, Nanning, China; 4 GuangDong Engineering Technology Research Center of Brain Function Assessment and Neuroregulation Rehabilitation, Guangzhou, China; 5 Institute of Exercise and Rehabilitation Science, Zhujiang Hospital, Southern Medical University, Guangzhou, China; 6 School of Sport Medicine and Physical Therapy, Beijing Sport University, Beijing, China; 7 School of Nursing, Southern Medical University, Guangzhou, China; 8 Department of Rehabilitation Sciences, The Hong Kong Polytechnic University, Hong Kong, China

**Keywords:** low back pain, artificial intelligence, exercise, telerehabilitation

## Abstract

**Background:**

Exercise therapy is strongly recommended as a treatment for chronic nonspecific low back pain (CNSLBP). However, therapist-guided exercise therapy requires significant medical resources. Ordinary digital telerehabilitation affects efficacy due to a lack of guidance and dynamic support. Artificial intelligence (AI)–assisted interactive health promotion systems may solve these problems.

**Objective:**

We aimed to explore whether AI-assisted multimodal exercise telerehabilitation is superior to conventional telerehabilitation in the treatment of people with CNSLBP.

**Methods:**

This study was a prospective, double-arm, open-label, randomized clinical controlled trial. People with CNSLBP were randomly allocated to either the AI or video group, receiving AI-assisted multimodal exercise therapy or conventional video guidance, respectively, via a WeChat application add-in. The multimodal exercise consisted of deep core muscle, flexibility, Mackenzie, and breathing exercises. The exercises were performed for 30‐45 minutes per session, 3 times a week, for 4 weeks. Participants underwent face-to-face assessment at baseline and week 4, and web-based assessment at weeks 2 and 8. The primary outcome was the change in Numerical Rating Scale (NRS) relative to baseline at week 4. Secondary outcomes included changes in the Roland-Morris Disability Questionnaire (RMDQ), Oswestry Disability Index (ODI), Pain Castastrophizing Scale (PCS), Timed Up-and-Go (TUG) test, and thickness of the transverse abdominus (TrA) and multifidus (MF) muscles relative to baseline at week 4. Generalized estimating equation and covariance were used to examine the efficacy of the interventions.

**Results:**

A total of 38 participants (19 participants per group) were recruited. Eighteen participants in the AI group and 16 participants in the video group completed and were included in the final analysis. There was a significant difference in NRS at week 4 between the AI group and video group (most severe NRS: −3.00 vs −1.50; adjusted mean difference −1.08, 95% CI −1.68 to −0.49; *P*<.001; mean NRS: −2.61 vs −1.62; adjusted mean difference −0.67, 95% CI −1.19 to −0.15; *P*=.01). The difference in most severe NRS persisted until week 8 (−3.06 vs −1.69; adjusted mean difference −0.95, 95% CI −1.73 to −0.18; *P*=.02). Compared with the video group at week 4, the AI group showed significant improvement in secondary outcomes, including RMDQ, PCS, and core muscle thickness of left TrA, right TrA, left MF, and right MF.

**Conclusions:**

We showed that 4 weeks of telerehabilitation based on AI-assisted multimodal exercise has better therapeutic effects compared to conventional exercise telerehabilitation in people with CNSLBP. This study provides guidance for developing effective real-time home-based exercise therapies for people with CNSLBP, which may help reduce economic and human resource costs associated with treatment.

## Introduction

Low back pain (LBP) is usually defined as pain between the costal margins and the subgluteal fold region [1], is one of the most common health problems worldwide, and causes a high socioeconomic impact [2]. The probability of developing LBP in a person’s lifetime is as high as 75%‐84% [3]. Pain lasting for more than 3 months is called chronic LBP (CLBP).

Studies have shown that 80%‐90% of CLBP is nonspecific and incurable without any specific pathological cause, which is called chronic nonspecific LBP (CNSLBP) [4]. In China, the incidence of CNSLBP is high. In the Departments of Orthopedics, Rehabilitation Medicine, and Pain Medicine, CNSLBP is the most common disease, which accounts for one-third of daily outpatient visits, second only to upper respiratory tract infections [5]. The high incidence makes a huge burden on the health care budget, which is greater than that of coronary heart disease, diabetes, arthritis, and cerebrovascular disease [6]. Thus, it is necessary to conduct in-depth research on CNSLBP and to determine improved treatment methods.

There are many current treatments for CNSLBP. Nonpharmacological therapies are the first-line treatment for CLBP [1]. Among these therapies, high-quality evidence suggests that exercise therapy is more effective in reducing pain compared to no treatment or other conservative treatments [7]. Exercise therapy, as an effective means of treating LBP, is not only effective but also cost-effective compared to other medical methods [8]. Therefore, clinical guidelines strongly recommend exercise therapy for people with CLBP [9].

There are various forms of exercise therapy, such as core stability training, Pilates, McKenzie exercises, breathing training, trunk muscle strength training, suspension training, and Tai Chi. To improve the effectiveness of exercise, specialists usually recommend using a combination of different conservative treatments [10]. In exercise therapy, the combination of flexibility and strength training (the basic composition of multimodal exercise) has been shown to be the most effective intervention for people with CLBP [11,12].

Exercise therapy can be practiced in medical institutions or at home. However, due to insufficient resources, it is difficult to carry out exercise therapy in medical institutions on a large scale. In most cases, exercise therapy is conducted through home-based training, which leads to problems such as a lack of guidance and dynamic support and difficulties in contacting care providers [13]. This reduces the effectiveness of this therapy. Therefore, exploring new home-based multimodal exercise training methods, which can offer feedback and guidance, is necessary.

The effectiveness of application-based therapy has been proven in exploring new home-based multimodal exercise training methods [14]. Using application-based therapy as a therapy supplement could help promote the implementation of home-based exercise protocols. The application features include exercise tracking of prefabricated or adaptable workout programs, educational aspects, artificial intelligence (AI)–based therapy, or workout programs, and motion detection [15]. In terms of AI-based applications, most previous research has focused on pushing personalized self-management to people with LBP [16-18], but this has not always achieved better therapeutic outcomes compared to conventional clinical care [17,18]. Few AI-based applications focused on motion detection and guidance. With the development of technology, AI human key point identification technology can accurately determine body surface key points and guide individuals during movement, which has been validated in knee or hip osteoarthritis [19]. However, there is no research on applying this technology to exercise therapy for CNSLBP.

Characterized by real-time guidance, simplification of instructions, and convenience of doctor-patient communication, multimodal exercise telerehabilitation based on AI human key point identification technology may improve the effectiveness of exercise therapy. However, studies regarding home-based multimodal exercise therapy based on AI human key point identification technology among people with CNSLBP are lacking. We hypothesized that AI-assisted exercise therapy would have a positive effect on therapeutic efficacy. We, therefore, aimed to explore the effects of AI-assisted multimodal exercise on pain intensity, body function, psychology, and the core muscles in CNSLBP telerehabilitation.

## Methods

### Trial Design

This was a prospective, double-arm, open-label, randomized controlled clinical study conducted in Guangzhou, China, from March to October 2023 at Zhujiang Hospital of Southern Medical University. All the participants were randomly assigned to either the AI-assisted exercise therapy group (AI group) or the video-assisted exercise therapy group (video group).

### Ethical Considerations

Protocol approval was obtained from the Ethics Committee of Zhujiang Hospital of Southern Medical University (number 2023-KY-017). The study was prospectively registered in the Chinese Clinical Trial Registry (registration number ChiCTR2300073185) and conducted in compliance with local policies and regulations. All participants were provided with an informed consent form based on the principles of the Helsinki Declaration, which included comprehensive details of the study. Participants were informed that they could withdraw from the study at any time, and the form also included clarifications on relevant issues to ensure they were fully informed. To protect privacy, the data of participants were anonymized using letter codes, and only the principal research team had access to this data. Participants were explicitly informed that their data would only be used for anonymous article publication. In the event of any adverse reactions, participants could contact the researchers through communication tools at any time. The researchers would then assess the situation to determine whether the participant should be withdrawn from the study and provided with alternative treatments, such as physical therapy or analgesics.

### Study Population

The sample size was determined by G*Power software (version 3.1.9.2; Kiel University). The effect size was determined based on a previous study using an AI-based application for CLBP exercise intervention [18]. In this study, the mean Numerical Rating Scale (NRS) of the exercise group decreased by 1.1 (SD 0.3) points. The mean NRS of conventional group decreased by 0.9 (SD 0.4) points. The average SD was 0.35. The effect size was calculated to be 0.57. The correlation between repeated measurements was set as 0.5. There were 2 groups with 4 measurements. With a statistical power of 0.95 and an α level of 0.05, the total sample size was calculated to be 28. Considering a 20% shedding rate, the recruitment target was at least 36 participants.

Inclusion criteria followed the nonspecific LBP diagnosis guidelines of the American College of Physicians and the American Pain Society [20]. Participants who met the following criteria were included in the study: (1) clinical diagnosis of nonspecific LBP or discomfort for >3 months, with a mean NRS≥3 points; (2) age 18 to 75 years; (3) right-handed; and (4) possession of a smartphone and the skill to operate WeChat. The key exclusion criteria were as follows: (1) pregnancy; (2) a history of waist trauma or waist or abdominal surgery in the past 2 years; (3) a history of nerve root symptoms, spine fracture, infection, lumbar malignancy, or LBP caused by any other disease; and (4) participants suffering from hypertension, heart disease, Parkinson’s disease, and other conditions that were not suitable for intense exercise. Participants were recruited through the pain management clinic of Zhujiang Hospital, WeChat friend circles, and recruitment posters.

All interested patients with LBP who presented for consultation were provided with basic information about the study, and a preliminary screening questionnaire was completed. The number of participants who were invited to participate, refused to participate, and were not eligible (and their related reasons) were recorded by a research assistant.

### Randomization and Blinding

Using a random number sequence generated by SPSS 20.0 software (IBM), all eligible participants were randomly assigned to the AI group or video group in a 1:1 ratio by a research assistant who was not involved in the assessments and treatment. Random numbers were hidden in opaque envelopes. Each envelope was successively numbered, and the screening number was attached to the surface. The research assistant opened the envelope to assign participants to the corresponding groups.

There were 6 people in the study team. A research assistant was responsible for the recruitment and another for the random assignment of participants. Two therapists with many years of treatment experience were responsible for the intervention exercises of the 2 groups. A rehabilitation physician was specifically responsible for the assessment of the outcomes. A statistical analyst was responsible for the statistical analysis. The statistical analyst and rehabilitation physician were blind to group allocation. As adequate informed consent was required, all participants were informed about what exercise intervention they would receive. Thus, this study was an open-label study.

### Intervention Measures

The intervention measures included 2 parts: patient education and exercise therapy [21]. Through the form of community management and graphic publicity, patient education could improve patient enthusiasm for exercise and provide participants with rehabilitation knowledge about correct posture and reasonable exercise modes. The multimodal exercises included deep core muscle [11,22], flexibility [23], Mackenzie [24], and breathing exercises [25] to improve patient core strength and increase joint flexibility, as shown in Multimedia Appendix 1.

The telerehabilitation of participants was realized through an application called Yun-fu (Beijing Yinshan Future Health Technology Co, Ltd.), a WeChat application add-in used for smartphones and tablets. The application add-in was used to collect patient evaluation information and complete the exercise training.

The angle and length ratios among the 30 key points of the human body were identified by AI for all the exercise movements involved, and were set in the application add-in to complete the standards and common errors of the movements. The specific procedures for participants using the application add-in were as follows. Participants opened the application add-in by scanning a QR code and completed the self-assessment, which included the basic information of CLBP, Oswestry Disability Index (ODI) questionnaire, STarT back questionnaire, Generalized Anxiety Disorder questionnaire, and the AI-assisted acquisition of lumbar spine mobility and core muscle endurance. According to the self-assessment results, the application add-in classified participants into 5 categories of LBP based on treatment-based classifications [26], including flexion intolerance, extension or rotation intolerance, stability deficiency, muscle tension, and nerve compression. Finally, the application add-in sent each patient exercise plans of 30‐45 minutes per session, 3 times a week, for a duration of 4 weeks [27]. In order to ensure consistency in the intervention exercise prescription between the 2 groups, the subjects included in this study were all subjects with LBP classified as stability deficiency.

**Figure 1.**
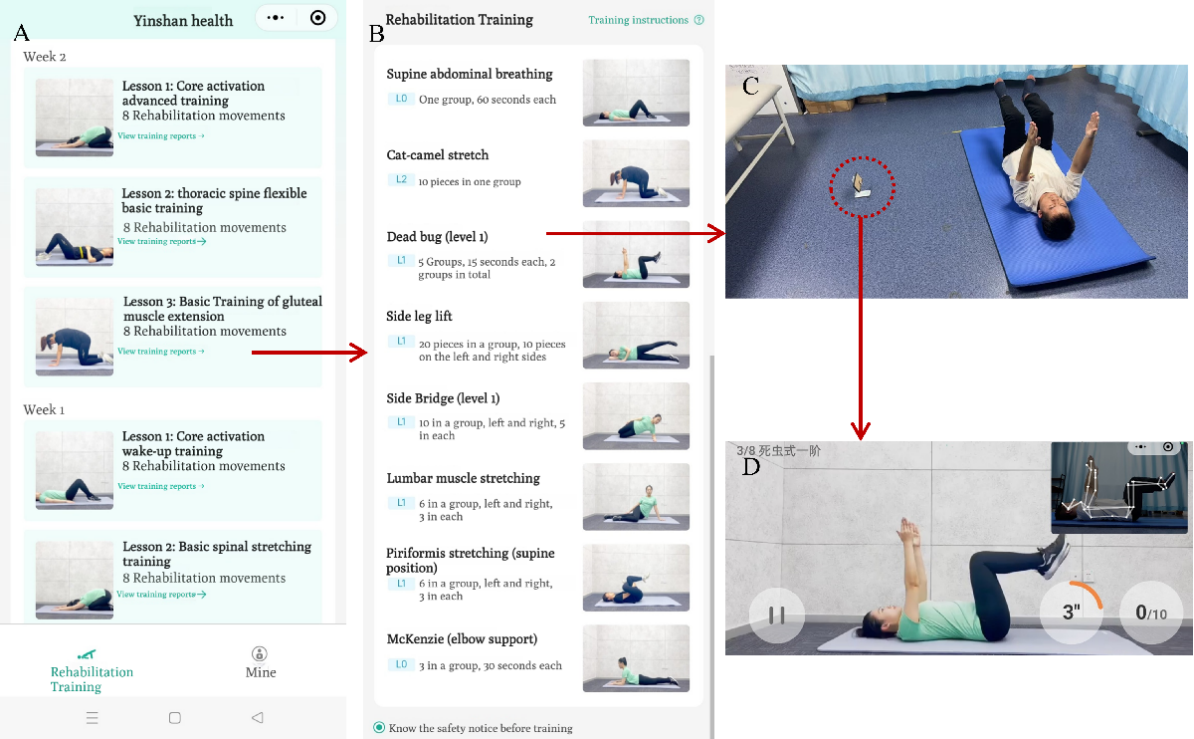
Example of artificial intelligence (AI)–assisted exercise in the Yunfu application add-in. (A) Weekly course schedule; (B) specific exercise prescriptions for each course; (C) scene arrangement during exercise; and (D) video and AI guidance on the mobile screen.

When the participants wanted to exercise, they simply needed to open the application add-in in WeChat (Figure 1A), select the corresponding prescription course (Figure 1B), stand or lie on the yoga mat, and place the phone on a phone stand 1.5 m away (Figure 1C). The application add-in would automatically broadcast the training content and movement points, and movement video demonstrations were presented on the screen. Furthermore, an AI recognition box in the upper right corner of the screen automatically recognized the movements and provided real-time guidance to ensure standardization of movements (Figure 1D). When a movement was complete, the application add-in immediately provided an evaluation and prompt, including “qualified,” “good,” “excellent,” and “super excellent,” to motivate participants to complete the following exercise.

After exercising, the application add-in sent a rating of perceived exertion and training reports. Participants then posted a screenshot of the training report in a prejoined WeChat group and received incentives from other participants and the rehabilitation therapists in the group. At the beginning of each week, participants received video and graphic education about CLBP from the therapists, including correct posture, pain management, causes of CLBP, and lumbar spine structure. At 2 and 4 weeks of exercise, participants completed the same initial assessment in the application add-in, and their program was appropriately adjusted.

The video group received the same education, evaluation, and exercise prescription as the AI group. The only difference was that, during the exercise process, the video group completed the exercise by watching a training video, and the AI group received training video and AI guidance during the process. The groups did not receive additional treatments throughout the entire study period.

### Outcomes and Assessments

Demographic information, including age, gender, BMI, years of education, and duration of pain (<6 mo, 6‐12 mo, >12 mo), was obtained using self-reported questionnaires. To describe the pain changes of participants more comprehensively, pain (current, mean, and most severe in the past week) was measured with the NRS [28]. The primary outcome of the study was the change in NRS relative to baseline at week 4. The secondary outcomes were changes in NRS at week 8, and the scores of ODI, Roland-Morris Disability Questionnaire (RMDQ), Pain Catastrophizing Scale (PCS), time of Timed Up-and-Go (TUG) test, and thickness of core muscles (transverse abdominus [TrA] and multifidus [MF]) at week 4, relative to baseline. The baseline and week 4 data were collected face-to-face in the laboratory. The remaining data were collected through web-based questionnaires.

The NRS uses a scale with numbers 0-10 to quantify pain intensity, with a higher number representing a greater degree of pain. A score of 0 indicates no pain; 1‐3, mild pain; 4‐6, moderate pain; and 7‐10, severe pain [29]. The RMDQ was developed to evaluate low back function with good reliability and validity [30]. The questionnaire has 24 options with a total score of 24 points. The higher the score, the greater the patient’s functional limitations. The ODI, comprising 10 questions evaluating pain intensity, daily activities, lifting, walking, sitting, standing, sleep, sexual life, social life, and travel, was used to assess the disability of participants. Each question has 6 options, corresponding to 0-5 points. Higher points represent more severe dysfunction [31]. The PCS was used to assess the catastrophic pain thoughts. The questionnaire consists of 13 items, rated on a 5-point scale (with 0=never, 1=almost never, 2=sometimes, 3=fairly often, and 4=very often). Higher scores reflect higher pain catastrophizing [32].

The TUG test was used to assess physical function. Participants began by sitting in a standard armchair with a height of approximately 46 cm. They were then asked to stand up from the armchair, walk 3 m at a comfortable speed, then immediately turn around, walk back to the armchair, and sit down again. The time was recorded between when they left the armchair and when they returned to a seated position [33]. The test was performed 3 times, and the results were averaged.

The muscle thickness of TrA and MF was measured by using a Terason uSmart 3300 ultrasound (Terason). TrA and MF are considered to be the most critical muscles in maintaining lumbar spine stability and are closely related to the development of LBP [34,35]. For the thickness of TrA, participants lay in the supine position with hips flexed to approximately 135° and knees flexed to 90°. They were then instructed to take a deep breath, exhale fully, and maintain a relaxed abdomen for 5 seconds. A linear probe was used to measure the thickness above the iliac crest in the anterior axillary line [36]. Muscle thickness was recorded as the distance between the upper and lower hyperechoic myofascial. For the thickness of MF, participants lay in the prone position, with a thin pillow under the abdomen to straighten the lumbar spine. The head of the participant was positioned in the treatment bed hole. A curved probe was used to measure the thickness at the fourth lumbar vertebra (L4). Muscle thickness was recorded as the distance from the tip of the articular process to the lower margin of subcutaneous fat [37]. The measurement was performed on the left and right sides, 3 times, with the average used for data analysis.

### Statistical Analysis

The mean (SD) or median (interquartile range) was used to describe continuous variables, whereas numbers (percentages) were used for categorical variables. All analyses were conducted based on the per-protocol principle. An independent *t*-test, Wilcoxon rank-sum test, or Fisher’s exact test was used to test the differences of baseline characteristics between groups, as appropriate. Normal distribution was examined by Shapiro-Wilk test and QQ plots. Analyses of variables changing from baseline at week 2 and week 4 (most severe NRS, mean NRS, current NRS, ODI, RMDQ, and PCS) were performed by generalized estimating equations and adjusted for the respective baseline value. Analyses of variables changing from baseline at week 8 (most severe NRS, mean NRS, and current NRS) were performed by generalized estimating equations and adjusted for the respective baseline value. Variables changing from baseline at week 4 (TUG and muscle thickness) were tested by covariance analysis adjusted for the respective baseline value. The significance level was set at *P*<.05 for all statistical tests. All analyses were performed in R (version 4.3.1; R Foundation for Statistical Computing) and SPSS (version 20.0; IBM).

## Results

### Study Population

**Figure 2.**
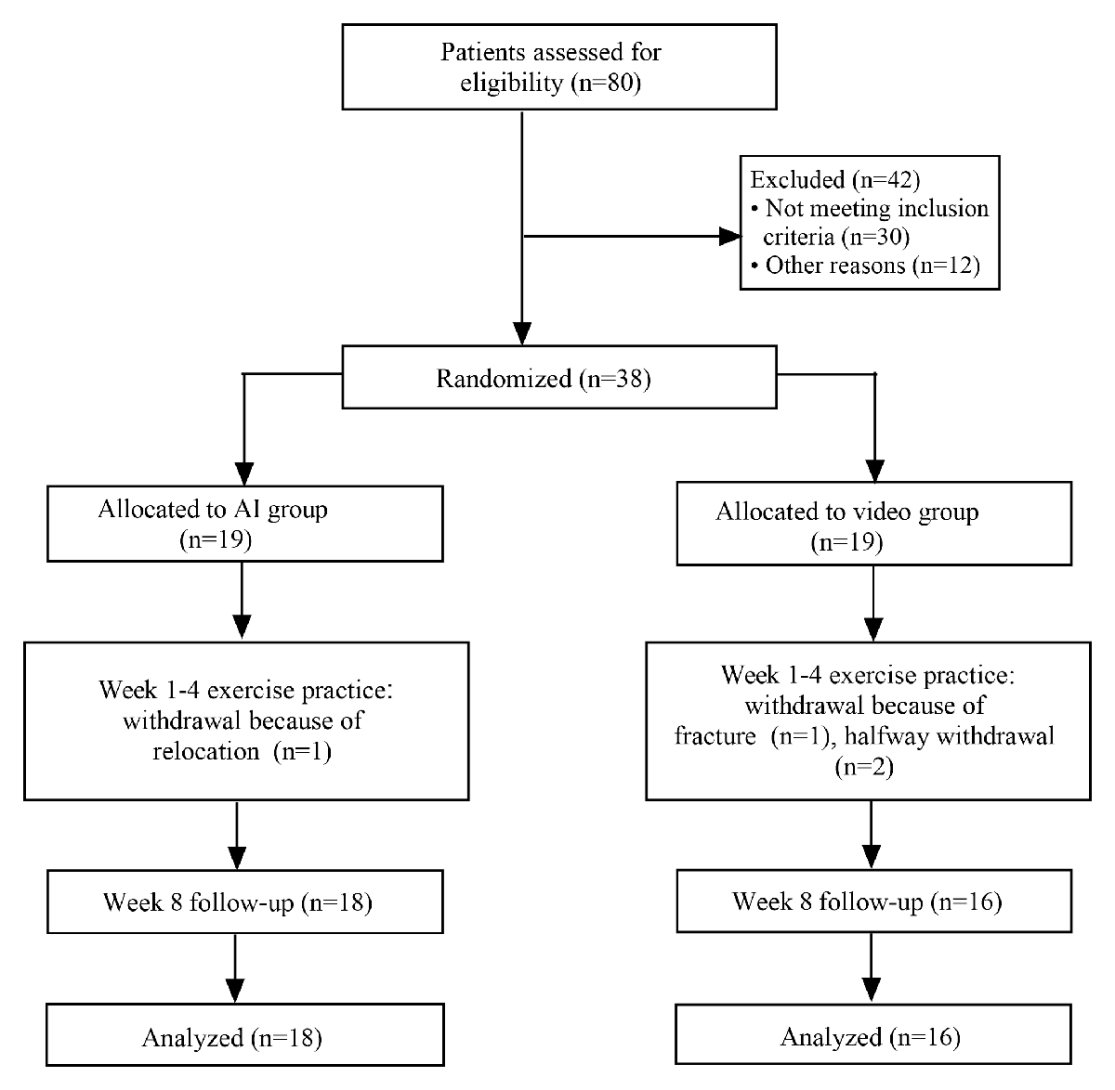
Flowchart of included participants. AI, artificial intelligence.

As shown in Figure 2, a total of 38 participants with CNSLBP were randomly assigned to either the AI or video group. Finally, 34 participants completed the intervention and follow-up (n=18, 47% analyzed for efficacy in the AI group; n=16, 42% analyzed for efficacy in the video group; Checklist 1).

Table 1 shows the baseline characteristics of participants included in the analysis. The mean age of the participants was 28.9 (SD 9.1) years and 29.3 (SD 7.4) years for the video and AI groups, respectively. Both groups were predominantly female (n=14 (88%) for the video group; n=12 (67%) for the AI group). The education duration of the video group was 17.0 (IQR 16.0-17.0) years, which was similar to the AI group with 16.0 (IQR 16.0-18.0) years. The 2 groups also had similar BMI results (21.0 (SD 2.9) kg/m^2^ for the video group; 21.3 (SD 2.4) kg/m^2^ for the AI group). A pain duration of 32.6 (SD 27.8) months was reported for the participants in the video group, which was less than that of the AI group, a pain duration of 53.3 (SD 61.2) months. These demographic results and baseline measurement data, including NRS, RMDQ, ODI, PCS, TUG, and thickness of core muscles (TrA and MF), showed no significant differences between the 2 groups.

**Table 1.** Baseline characteristics.

Characteristic	Statistic	Video group(n=16)	AI^a^ group(n=18)	*P* value
Age (years)	Mean (SD)	28.9 (9.1)	29.3 (7.4)	.91
Gender				.23
Male	n (%)	2 (13%)	6 (33%)	
Female	n (%)	14 (88%)	12 (67%)	
Education (years)	Median (IQR)	17.0 (16.0-17.0)	16.0 (16.0-18.0)	.91
BMI (kg/m^2^)	Mean (SD)	21.0 (2.9)	21.3 (2.4)	.78
Pain duration (months)	Mean (SD)	32.6 (27.8)	53.3 (61.2)	.21
Most severe NRS^b^	Median (IQR)	4.0 (4.0-5.0)	5.0 (4.0-5.8)	.15
Mean NRS	Median (IQR)	4.0 (3.0-4.0)	4.0 (3.0-5.0)	.21
Current NRS	Median (IQR)	3.0 (3.0-4.0)	3.0 (3.0-3.8)	.83
ODI^c^	Mean (SD)	7.8 (2.8)	8.4 (3.1)	.50
RMDQ^d^	Mean (SD)	4.5 (2.0)	5.1 (1.7)	.39
PCS^e^	Mean (SD)	13.9 (9.1)	18.6 (9.1)	.14
Timed up-and-go (s)	Mean (SD)	10.2 (1.2)	10.4 (1.1)	.69
LTrA^f^ thickness (mm)	Mean (SD)	3.8 (0.5)	4.1 (0.8)	.19
RTrA^g^ thickness (mm)	Mean (SD)	3.9 (0.6)	4.0 (0.6)	.56
LMF^h^ thickness (mm)	Mean (SD)	26.8 (3.2)	27.4 (4.1)	.67
RMF^i^ thickness (mm)	Mean (SD)	25.7 (3.3)	26.6 (4.4)	.50

a AI, artificial intelligence.

b NRS, Numerical Rating Scale.

c ODI, Oswestry Disability Index.

d RMDQ, Roland-Morris Disability Questionnaire.

e PCS, Pain Catastrophizing Scale.

f LTrA, left transverse abdominal muscle.

g RTrA, right transverse abdominal muscle.

h LMF, left multifidus muscle.

i RMF, right multifidus muscle.

### Changes in NRS

**Figure 3.**
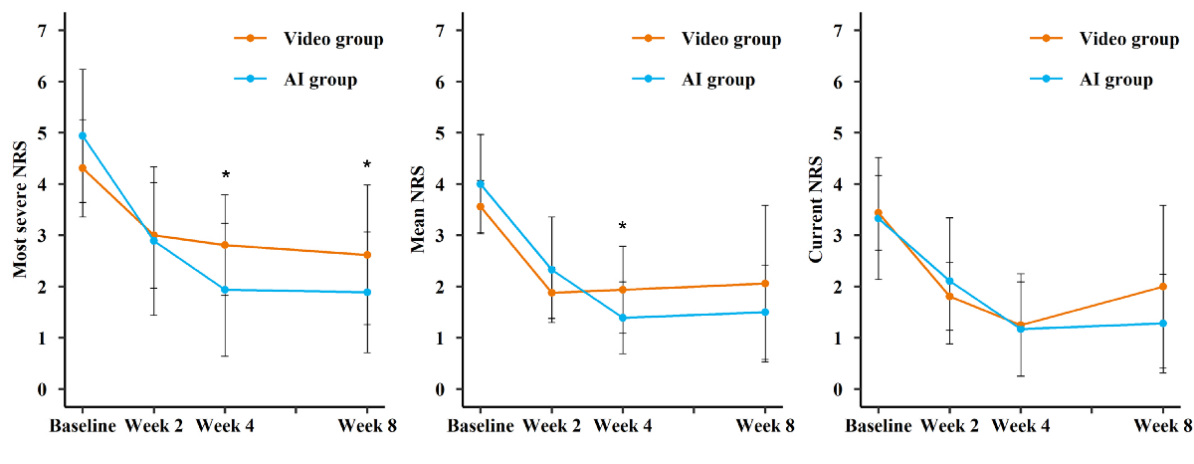
Numerical Rating Scale (NRS) at baseline and changes in 2 weeks, 4 weeks, and 4 weeks of follow-up. AI, artificial intelligence.

At week 4, the mean change (SD) in the most severe NRS in the AI group and video group was −3.00 (SD 1.41) and −1.50 (SD 1.03), respectively (adjusted mean difference −1.08, 95% CI −1.68 to −0.49; *P*<.001). The mean change in mean NRS in the AI group and video group was −2.61 (SD 0.92) and −1.62 (SD 0.96), respectively (adjusted mean difference −0.67, 95% CI −1.19 to −0.15; *P*=.01). There was no significant difference between the AI group (mean −2.17, SD 1.29) and video group (mean −2.19, SD 1.22; adjusted mean difference −0.07, 95% CI −0.71 to 0.57; *P*=.83) in current NRS. At week 2, no significant difference in the reduction of NRS (most severe, mean, and current) was observed between the 2 groups. At follow-up of week 8, the change in the most severe NRS in the AI group and video group was −3.06, SD 1.51, and −1.69, SD 1.54, respectively (adjusted mean difference −0.95, 95% CI −1.73 to −0.18; *P*=.02). No significant difference was observed in mean NRS and current NRS between the 2 groups (Table 2). The 3-dimensional NRS changes between the AI group and the video group during the study period are shown in Figure 3.

**Table 2.** Primary and secondary outcomes change from baseline.

Outcome	AI^a^ group, mean (SD)	Video group, mean (SD)	Adjusted mean difference(95% CI)	*P* value^b^
Most severe NRS^c^				
Week 2	−2.06 (1.47)	−1.31 (1.30)	−0.33 (−1.05 to 0.40)	.38
Week 4	−3.00 (1.41)	−1.50 (1.03)	−1.08 (−1.68 to −0.49)	<.001
Week 8	−3.06 (1.51)	−1.69 (1.54)	−0.95 (−1.73 to −0.18)	.02
Mean NRS				
Week 2	−1.67 (1.19)	−1.69 (0.79)	0.34 (−0.19 to 0.87)	.20
Week 4	−2.61 (0.92)	−1.62 (0.96)	−0.67 (−1.19 to −0.15)	.01
Week 8	−2.50 (1.10)	−1.50 (1.55)	−0.68 (−1.53 to 0.17)	.12
Current NRS				
Week 2	−1.22 (1.63)	−1.62 (1.15)	0.31 (−0.34 to 0.96)	.35
Week 4	−2.17 (1.29)	−2.19 (1.22)	−0.07 (−0.71 to 0.57)	.83
Week 8	−2.06 (1.11)	−1.88 (1.86)	−0.27 (−1.15 to 0.60)	.54
Timed up-and-go (s)				
Week 4	−1.05 (0.78)	−0.85 (0.86)	−0.15 (-0.65 to 0.35)	.55
Left TrA^d^ thickness (mm)				
Week 4	0.30 (0.45)	−0.14 (0.31)	0.47 (0.19 to 0.75)	.002
Right TrA thickness (mm)				
Week 4	0.57 (0.41)	−0.09 (0.47)	0.69 (0.39 to 0.99)	<.001
Left MF^e^ thickness (mm)				
Week 4	0.90 (1.15)	−0.01 (1.11)	0.94 (0.14 to 1.74)	.02
Right MF thickness (mm)				
Week 4	0.84 (0.85)	−0.03 (0.98)	0.92 (0.27 to 1.56)	.007
ODI^f^				
Week 2	−2.06 (2.21)	−1.56 (3.16)	−0.16 (−1.66 to 1.34)	.83
Week 4	−3.67 (2.59)	−2.25 (1.69)	−1.09 (−2.26 to 0.09)	.07
RMDQ^g^				
Week 2	−2.39 (2.23)	−2.00 (1.71)	−0.01 (−0.98 to 0.96)	.99
Week 4	−3.17 (1.82)	−1.88 (1.02)	−0.91 (−1.48 to −0.34)	.002
PSC^h^				
Week 2	−4.33 (7.77)	−1.12 (6.83)	−1.65 (−6.14 to 2.84)	.47
Week 4	−9.06 (6.80)	−3.56 (6.15)	−3.94 (−7.69 to −0.19)	.04

a AI, artificial intelligence.

b The significance level was set at *P*<.05

c NRS, Numerical Rating Scale.

d TrA, transverse abdominal muscle.

e MF, multifidus muscle.

f ODI, Oswestry Disability Index.

g RMDQ, Roland-Morris Disability Questionnaire.

h PCS, Pain Catastrophizing Scale. Each outcome variable was analyzed after adjusting the baseline value.

### Changes in RMDQ

**Figure 4.**
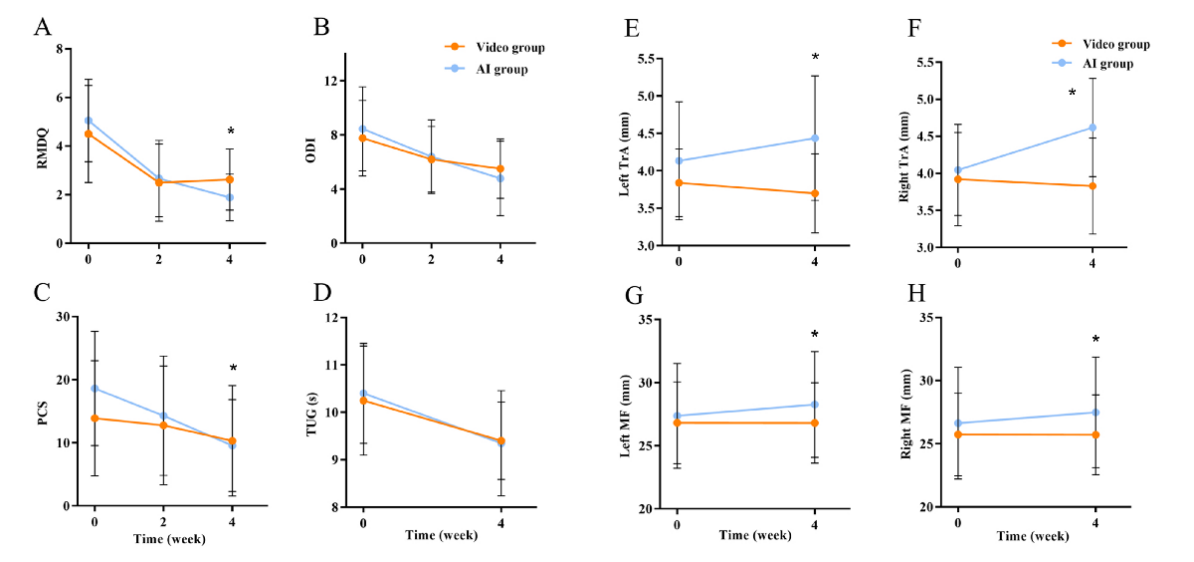
Secondary outcomes in baseline and changes from baseline to 4 weeks. (A) RMDQ (Roland-Morris Disability Questionnaire); (B) ODI (Oswestry Disability Index); (C) PCS (Pain Catastrophizing Scale); (D) TUG (Timed Up-and-Go); (E) left TrA (transverse abdominus); (F) right TrA; (G) left MF (multifidus); (H) right MF.

Compared with the baseline, the RMDQ scores of the AI group and video group showed a downward trend at week 2 and week 4 (Figure 4A). A significant difference was observed in the AI group (mean −3.17, SD 1.82) compared with that in the video group (mean −1.88, SD 1.02; adjusted mean difference −0.91, 95% CI −1.48 to −0.34; *P*=.002) at week 4. There was no significant difference between the AI group (mean −2.39, SD 2.23) and video group (mean −2.00, SD 1.71; adjusted mean difference −0.01, 95% CI −0.98 to 0.96; *P*=.99) at week 2 (Table 2).

### Changes in ODI

The ODI scores of the AI group and video group showed a downward trend at week 2 and week 4 compared with the baseline (Figure 4B). At week 4, no significant difference was observed in the AI group (mean −3.67, SD 2.59) and video group (mean −2.25, SD 1.69); adjusted mean difference −1.09, 95% CI −2.26 to 0.09; *P*=.07). Similarly, there was no significant difference between the AI group (mean −0.26, SD 2.21) and video group (mean −1.56, SD 3.16); adjusted mean difference −0.16, 95% CI −1.66 to 1.34; *P*=.83) at week 2 (Table 2).

### Changes in PCS

An improvement of PCS was observed at week 2 and week 4 compared with the baseline (Figure 4C). There was a significant difference between the AI group (mean −9.06, SD 6.80) and video group (mean −3.56, SD 6.15; adjusted mean difference −3.94, 95% CI −7.69 to −0.19; *P*=.04) at week 4. However, no significant difference was observed between the 2 groups (AI, mean −4.33, SD 7.77; video, mean −1.12, SD 6.83; adjusted mean difference −1.65, 95% CI −6.14 to 2.84; *P*=.47) at week 2 (Table 2).

### Changes in TUG

Compared with the baseline, the TUG test time for both groups decreased (Figure 4D). No difference was observed in the AI group (mean −1.05, SD 0.78) and video group (mean −0.85, SD 0.86; adjusted mean difference −0.15, 95% CI −0.65 to 0.35; *P*=.55) at week 4 (Table 2).

### Changes in Core Muscle Thickness

Figure 4E–H shows the thickness changes of core muscles (TrA and MF) in the 2 groups throughout the study period. At week 4, significant differences were observed for the thickness of TrA and MF on both sides between the AI group and video group (left TrA: 0.30 vs −0.14; adjusted mean difference 0.47, 95% CI 0.19 to 0.75; *P*=.002; right TrA: 0.57 vs −0.09; adjusted mean difference 0.69, 95% CI 0.39 to 0.99; *P*<.001; left MF: 0.90 vs −0.01; adjusted mean difference 0.94, 95% CI 0.14 to 1.74; *P*=.02; right MF: 0.84 vs −0.03; adjusted mean difference 0.92, 95% CI 0.27 to 1.56; *P*=.007) (Table 2).

## Discussion

### Principal Findings

This study examined the therapeutic effect of AI real-time guidance exercise therapy compared with conventional video guidance exercise therapy in young people with CNSLBP. We found that after 4 weeks of AI-assisted exercise therapy, the pain intensity, body function, psychology, and core muscle thickness had higher improvement compared to conventional exercise therapy.

### The Efficacy of AI-Assisted Exercise Telerehabilitation for Participants With CNSLBP

Exercise therapy improves symptoms in participants with CNSLBP. However, as an active exercise and mostly in the form of telerehabilitation, exercise therapy has the problems of insufficient compliance, an inability to receive guidance, and reduced efficacy [13]. AI-based telemedicine has been expected to solve these challenges. After 4 weeks of exercise, the NRS showed a downward trend in 2 groups. Furthermore, the change of most severe NRS and mean NRS in the AI group was significantly different compared to the conventional video group, suggesting that AI-assisted exercise therapy could reduce pain to a greater extent. There have been several previous similar studies based on AI-based exercise therapy for CLBP [16-18,38,39], but not characterized by motion detection and guidance. Some of these studies have shown similar results in pain as our research [38,39]. The comparison demonstrates that AI-based telerehabilitation focusing on motion detection and guidance is feasible in the treatment of CNSLBP. Moreover, a previous study has established that a minimal clinical important difference (MCID) on the NRS is 2/10 points [40]. In our study, the AI group reached MCID at week 2 on the most severe NRS and reached MCID at week 4 on the mean NRS and current NRS, while the video group only reached MCID on the current NRS at week 4. As we know, MCID is not a statistically significant difference, but it is an indicator of the clinical benefits to patients. The difference in the time to achieve MCID reflects that AI-assisted exercise training has better therapeutic effects compared to traditional training. The MCID of the AI group still existed at the week 8 follow-up, while the video group did not reach the MCID, further reflecting the efficacy stability of AI-assisted exercise training. People with CNSLBP received standardized exercise training through AI guidance and increased their motivation to engage in exercise training through real-time feedback, which we believe is the reason for the greater pain reduction in the AI group.

AI-assisted exercise training could improve the physical function of participants with CNSLBP more than traditional training. For physical function in the secondary outcomes, we used ODI and RMDQ for subjective evaluation and used the TUG test for objective evaluation. All 3 indicators showed a downward trend at week 4. Only RMDQ showed a significant difference between the 2 groups. A previous study has shown that RMDQ is more suitable for people with mild to moderate disability, and ODI suitable for people with severe disability [41]. In our study, the majority of participants with CNSLBP were middle-aged and young people with mild to moderate disability, they were still in the early stage of LBP, which are the reasons why only RMDQ showed a significant difference. A previous study has established that an MCID on RMDQ is a 30% change from baseline [42]. Both groups reached MCID on RMDQ at week 2 and week 4, but the change amplitude of the AI group was greater, suggesting that AI-assisted exercise training was superior in certain aspects in reducing physical disabilities. The result is consistent with a previous study [16].

Previous theory has shown that catastrophizing psychology is a prerequisite for the adverse outcomes of chronic musculoskeletal pain [43]. Amplifying pain perception when faced with chronic musculoskeletal pain will increase pain sensitivity, ultimately leading to further exacerbation of pain. Thus, improving catastrophic psychology is equally important for the treatment of CNSLBP. In our study, both groups showed a downward trend on PCS. A previous study investigated the effects of exercise therapy on catastrophizing psychology in people with CNSLBP. The results indicated that 8 weeks of core stability training could effectively improve catastrophizing psychology [44], which is consistent with our findings. Moreover, there was a significant difference between the 2 groups, suggesting that AI-assisted exercise training could better improve the catastrophizing psychology of people with CNSLBP.

Different from previous studies, we measured the thickness of TrA and MF at the end of the 4 weeks of exercise therapy. The occurrence of LBP is usually related to the morphological changes of TrA and MF. These changes usually manifest as fat infiltration, abnormal changes in type I and type II fibers, and asymmetric muscle atrophy [45]. Compared to normal individuals, a decrease in the 2 muscles’ thickness can be observed through imaging examinations among people with CNSLBP [46,47]. In our study, AI-assisted exercise therapy significantly increased the thickness of the 2 muscles, while the results of the video group did not show any significant changes. A previous study investigated the effect of routine remote core stability training over 4 weeks on TrA, and the results showed that there was no significant change in muscle thickness [48]. However, 2 other studies with 4 weeks of core stability training guided by therapists showed significant changes in the thickness of TrA and MF [36,49]. We thought that the reason for this difference was the presence of timely guidance. In our study, AI human key point identification technology replaced therapists to present real-time guidance and feedback. To be precise, guided exercise therapy, rather than simple exercise therapy, has been recommended as the first-line treatment for CNSLBP [50].

To our knowledge, this is the first study to apply AI human key point identification technology to specific exercise training movements among participants with CNSLBP. Through timely guidance of exercise therapy, participants can accurately exercise the target core muscle groups, enhance core strength, increase spinal stability, and ultimately alleviate CNSLBP. Overall, our study demonstrated the effectiveness of AI-assisted exercise therapy compared to conventional therapy from subjective and objective perspectives.

### Limitations

Our study had some limitations. First, most of our participants had mild to moderate pain, whereas few had severe pain. The efficacy of AI-assisted exercise therapy for severe LBP is still unclear. Second, we only conducted 4 weeks of follow-up, with lasting efficacy. Sustained efficacy beyond this period remains to be explored. Finally, our participants were middle-aged or young. Whether our findings could be extrapolated to older adults is unknown.

### Conclusion

To address the limitations of a lack of guidance and dynamic support in conventional home-based exercise therapy telerehabilitation, we explored a new treatment based on AI human key point identification technology. We found that AI-assisted multimodal exercise therapy had better therapeutic effects compared to conventional telerehabilitation on pain intensity, body function, psychology, and the core muscles. In the future management of CNSLBP, further consideration can be given to applying this treatment model to maximize the effectiveness of telerehabilitation.

## Supplementary material

10.2196/56176 Multimedia Appendix 1 Exercise prescription list.

10.2196/56176 Checklist 1 CONSORT-EHEALTH checklist (V 1.6).

## Abbreviations

AI: artificial intelligence
CLBP: chronic low back pain
CNSLBP: chronic nonspecific low back pain
LBP: low back pain
MCID: minimal clinical important difference
MF: multifidus
NRS: Numerical Rating Scale
ODI: Oswestry Disability Index
PCS: Pain Catastrophizing Scale
RMDQ: Roland-Morris Disability Questionnaire
TrA: transverse abdominus
TUG: Timed Up-and-Go
