# Correction: Clinical Efficacy of Multimodal Exercise Telerehabilitation Based on AI for Chronic Nonspecific Low Back Pain: Randomized Controlled Trial

**DOI:** 10.2196/78188

**Published:** 2025-06-06

**Authors:** Chongwu Xiao, Yijin Zhao, Gege Li, Zhuodong Zhang, Siyu Liu, Weichao Fan, Jinjing Hu, Qiuru Yao, Chengduan Yang, Jihua Zou, Qing Zeng, Guozhi Huang

**Affiliations:** 1 Center for Rehabilitation Medicine, Zhujiang Hospital, Southern Medical University, No.253, Industrial Avenue Middle Guangzhou, Guangzhou, 510280, China, 86 19543576136; 2 School of Rehabilitation Sciences, Southern Medical University, Guangzhou, China; 3 Department of Rehabilitation Medicine, The Second Affiliated Hospital of Guangxi Medical University, Guangxi Medical University, Nanning, China; 4 GuangDong Engineering Technology Research Center of Brain Function Assessment and Neuroregulation Rehabilitation, Guangzhou, China; 5 Institute of Exercise and Rehabilitation Science, Zhujiang Hospital, Southern Medical University, Guangzhou, China; 6 School of Sport Medicine and Physical Therapy, Beijing Sport University, Beijing, China; 7 School of Nursing, Southern Medical University, Guangzhou, China; 8 Department of Rehabilitation Sciences, The Hong Kong Polytechnic University, Hong Kong, China

In “Clinical Efficacy of Multimodal Exercise Telerehabilitation Based on AI for Chronic Nonspecific Low Back Pain: Randomized Controlled Trial” (JMIR Mhealth Uhealth 2025;13:e56176) the authors noted one omission.

In the Acknowledgements, the following sentence has been added:

Jihua Zou, Qing Zeng, and Guozhi Huang are corresponding authors and contributed equally to this work.

The correction will appear in the online version of the paper on the JMIR Publications website, together with the publication of this correction notice. Because this was made after submission to PubMed, PubMed Central, and other full-text repositories, the corrected article has also been resubmitted to those repositories.

