# Supplementary material for: Clinical Efficacy of Multimodal Exercise Telerehabilitation Based on AI for Chronic Nonspecific Low Back Pain: Randomized Controlled Trial
Source: JMIR Mhealth Uhealth. 2025 May 22;13:e56176. doi: 10.2196/56176 (PMC12121543; doi:10.2196/56176)
Supplement: Checklist 1 [file mhealth-v13-e56176-s002.pdf]

CONSORT-EHEALTH Checklist V1.6 Report	Manuscript Number	56176

(based on CONSORT-EHEALTH V1.6), available at [http://tinyurl.com/consort-ehealth-v1-6].

Date completed
9/1/2024 11:17:07
by
Chongwu *Xiao*

Clinical Efficacy of Multimodal Exercise Telerehabilitation Based on Artificial Intelligence for Chronic Non-specific Low Back Pain: A Randomized Controlled Trial

TITLE

1a-i) Identify the mode of delivery in the title
"Telerehabilitation Based on Artificial Intelligence" in the title
1a-ii) Non-web-based components or important co-interventions in title

1a-iii) Primary condition or target group in the title
"for Chronic Non-specific Low Back Pain"

ABSTRACT

1b-i) Key features/functionalities/components of the intervention and comparator in the METHODS section of the ABSTRACT
"AI-assisted multimodal exercise therapy via a WeChat application addin,"
1b-ii) Level of human involvement in the METHODS section of the ABSTRACT

1b-iii) Open vs. closed, web-based (self-assessment) vs. face-to-face assessments in the METHODS section of the ABSTRACT
"Participants underwent face-to-face assessment at baseline and week 4, and online assessment at week 2 and 8. "
1b-iv) RESULTS section in abstract must contain use data
"18 participants in AI group and 16 participants in *V*ideo group completed and were included in the final analysis. "
1b-v) CONCLUSIONS/DISCUSSION in abstract for negative trials

INTRODUCTION

2a-i) Problem and the type of system/solution
" In most cases, exercise therapy is conducted through home-based training, which leads to problems such as a lack of guidance and dynamic support and difficulties in contacting care providers. This reduces the effectiveness of this therapy. Therefore, exploring new home-based multimodal exercise training methods, which can offer feedback and guidance, is necessary."
2a-ii) Scientific background, rationale: What is known about the (type of) system
" With the development of technology, AI human key point identification technology can accurately determine body surface key points and guide individuals during movement, which has been validated in Knee or Hip Osteoarthritis. However, there is no research on applying this technology to exercise therapy for CNSLBP."
Does your paper address CONSORT subitem 2b?

"We hypothesized that AI-assisted exercise therapy would have a positive effect on therapeutic efficacy. We, therefore, aimed to explore the effects of AI-assisted multimodal exercise on pain intensity, body function, psychology, and the core muscles in CNSLBP telerehabilitation.

METHODS

3a) CONSORT: Description of trial design (such as parallel, factorial) including allocation ratio
"This was a prospective, double-arm, open label, randomized clinical study conducted in Guangzhou, China from March to October 2023 at Zhujiang Hospital of Southern Medical University."
3b) CONSORT: Important changes to methods after trial commencement (such as eligibility criteria), with reasons
No significant changes in methodology have occurred since the start of this study
3b-i) Bug fixes, Downtimes, Content Changes

4a) CONSORT: Eligibility criteria for participants
"Inclusion criteria:
□clinical diagnosis of non-specific LBP or discomfort for >3 months;
□Numerical Rating Scale (NRS) ≥3 points;
□age 18 to 75 years;
□right-handed;
□possession of a smartphone;
□having the skill to operate WeChat;
Exclusion criteria:
□pregnancy;
□a history of waist trauma or waist/abdominal surgery in the past 2 years;
□a history of nerve roots symptoms, spine fracture, infection, lumbar malignancy;
□LBP caused by any other disease;
□participants suffering from hypertension, heart disease, Parkinson's disease, and other conditions that were not suitable for intense exercise."
4a-i) Computer / Internet literacy
"□possession of a smartphone and the skill to operate WeChat."
4a-ii) Open vs. closed, web-based vs. face-to-face assessments:
"Participants were recruited through the pain management clinic of Zhujiang Hospital, WeChat friend circles, and recruitment posters."
4a-iii) Information giving during recruitment
"All interested patients with LBP who presented for consultation were provided with basic information about the study, and a preliminary screening questionnaire was completed. "
4b) CONSORT: Settings and locations where the data were collected
"The baseline and week 4 data were collected face-to-face in laboratory. The remaining data was collected through online questionnaires."
4b-i) Report if outcomes were (self-)assessed through online questionnaires
"The baseline and week 4 data were collected face-to-face in laboratory. The remaining data was collected through online questionnaires."
4b-ii) Report how institutional affiliations are displayed

5) CONSORT: Describe the interventions for each group with sufficient details to allow replication, including how and when they were actually administered
5-i) Mention names, credential, affiliations of the developers, sponsors, and owners

5-ii) Describe the history/development process

5-iii) Revisions and updating

5-iv) Quality assurance methods

5-v) Ensure replicability by publishing the source code, and/or providing screenshots/screen-capture video, and/or providing flowcharts of the algorithms used

5-vi) Digital preservation

5-vii) Access

" Participants opened the application addin by scanning a QR code, and completed the self-assessment ,the application addin sent each patient exercise plans of 30 – 45 minutes per session, 3 times a week, for a duration of 4 weeks"

5-viii) Mode of delivery, features/functionalities/components of the intervention and comparator, and the theoretical framework

"Participants opened the application addin by scanning a QR code, and completed the self-assessment. According to the self-assessment results, the application addin classified participants into five categories of LBP based on treatment-based classifications, including flexion intolerance, extension/ rotation intolerance, stability deficiency, muscle tension, and nerve compression. Finally, the application addin sent each patient exercise plans of 30 – 45 minutes per session, 3 times a week, for a duration of 4 weeks."

5-ix) Describe use parameters

"the application addin sent each patient exercise plans of 30 – 45 minutes per session, 3 times a week, for a duration of 4 weeks."

5-x) Clarify the level of human involvement

At the beginning of each week, participants received video and graphic education about CLBP from the therapists, including correct posture, pain manage ment, causes of CLBP, lumbar spine structure.

5-xi) Report any prompts/reminders used

"After exercising, the application addin sent rating of perceived exertion and training reports. Participants then posted a screenshot of the training report in a prejoined WeChat group, and received incentives from other participants and the rehabilitation therapists in the group. "

5-xii) Describe any co-interventions (incl. training/support)

"The video group received the same education, evaluation, and exercise prescription as the AI group,completed the exercise by watching a training video"

6a) CONSORT: Completely defined pre-specified primary and secondary outcome measures, including how and when they were assessed

"The primary outcome of the study was the change in NRS relative to baseline at week 4. The secondary outcomes were changes in NRS at week 8, and the scores of ODI, Roland – Morris Disability Questionnaire (RMDQ), Pain Castastrophizing Scale (PCS), time of Timed Up-and-Go (TUG) test, and thickness of core muscles (TrA and MF) at week 4, relative to baseline."

6a-i) Online questionnaires: describe if they were validated for online use and apply CHERRIES items to describe how the questionnaires were designed/deployed

6a-ii) Describe whether and how "use" (including intensity of use/dosage) was defined/measured/monitored

6a-iii) Describe whether, how, and when qualitative feedback from participants was obtained

6b) CONSORT: Any changes to trial outcomes after the trial commenced, with reasons

" The baseline and week 4 data were collected face-to-face in laboratory. The remaining data was collected through online questionnaires."

7a) CONSORT: How sample size was determined

7a-i) Describe whether and how expected attrition was taken into account when calculating the sample size

"The sample size was determined by G*Power software (version 3.1.9.2, Kiel University, Kiel, Germany). The effect size was determined based on a previous study using an AI-based application for CLBP exercise intervention [18]. In this study, the mean Numerical Rating Scale (NRS) of the exercise group decreased by 1.1 points, the standard deviation (SD) was 0.3. The mean NRS of conventional group decreased by 0.9 points, the SD was 0.4. The average SD was 0.35. The effect size was calculated to be 0.57. The correlation between repeated measurement was set as 0.5. There were two groups with four measurements. With a statistical power of 0.95 and an α level of 0.05, the total sample size was calculated to be 28. Considering a 20% shedding rate, the recruitment target was at least 36 participants."

7b) CONSORT: When applicable, explanation of any interim analyses and stopping guidelines

"The primary outcome of the study was the change in NRS relative to baseline at week 4. The secondary outcomes were changes in NRS at week 8, and the scores of ODI, Roland – Morris Disability Questionnaire (RMDQ), Pain Castastrophizing Scale (PCS), time of Timed Up-and-Go (TUG) test, and thickness of core muscles (TrA and MF) at week 4, relative to baseline."

8a) CONSORT: Method used to generate the random allocation sequence

"all eligible participants were randomly assigned to AI group or video group in a 1:1 ratio by a research assistant who was not involved in the assessments and treatment."

8b) CONSORT: Type of randomisation; details of any restriction (such as blocking and block size)

"Random numbers were hidden in opaque envelopes. Each envelope was successively numbered, and screening number was attached to the surface. "

9) ORT: Mechanism used to implement the random allocation sequence (such as sequentially numbered containers), describing any steps taken to conceal the sequence until interventions were assigned

Random numbers were hidden in opaque envelopes.

10) CONSORT: Who generated the random allocation sequence, who enrolled participants, and who assigned participants to interventions

"A research assistant was responsible for the recruitment, and another for the random assignment of participants.
involved in the intervention"

11a) CONSORT: Blinding - If done, who was blinded after assignment to interventions (for example, participants, care providers, those assessing outcomes) and how

11a-i) Specify who was blinded, and who wasn't

"The statistical analyst and rehabilitation physician were blind to group allocation. "

11a-ii) Discuss e.g., whether participants knew which intervention was the "intervention of interest" and which one was the "comparator"

11b) CONSORT: If relevant, description of the similarity of interventions

"The video group received the same education, evaluation, and exercise prescription as the AI group. "

12a) CONSORT: Statistical methods used to compare groups for primary and secondary outcomes

"Analyses of variables changing from baseline at week 2 and 4 (NRS, ODI, RMDQ, and PCS) were performed by generalized estimating equations and adjusted for the respective baseline value. Analyses of variables changing from baseline at week 8 (NRS) were performed by generalized estimating equations and adjusted for the respective baseline value. Variables changing from baseline at week 4 (TUG and muscle thickness) were tested by covariance analysis adjusted for the respective baseline value. The significance level was set at P <.05 for all statistical tests."

12a-i) Imputation techniques to deal with attrition / missing values

"All analyses were conducted based on the per-protocol principle."

12b) CONSORT: Methods for additional analyses, such as subgroup analyses and adjusted analyses

Each outcome variable was analyzed after adjusting the baseline value, and the dropping rate was within the 20% allowed.

RESULTS

13a) CONSORT: For each group, the numbers of participants who were randomly assigned, received intended treatment, and were analysed for the primary outcome

"34 participants completed the intervention and follow-up"

13b) CONSORT: For each group, losses and exclusions after randomisation, together with reasons

"Figure 2. Flowchart of included participants"

13b-i) Attrition diagram

"Figure 2. Flowchart of included participants"

14a) CONSORT: Dates defining the periods of recruitment and follow-up

"From March to October 2023 at Zhujiang Hospital of Southern Medical University, 80 patients were considered for eligibility. "

14a-i) Indicate if critical "secular events" fell into the study period

14b) CONSORT: Why the trial ended or was stopped (early)

Our study did not end early		
**15) CONSORT: A table showing baseline demographic and clinical characteristics for each group**		
"The population was randomly allocated into two groups: AI group (n = 19) and *Video group* (n = 19), as illistrated in Figure 2."		
**15-i) Report demographics associated with digital divide issues**		
"Table 1 Baseline characteristics"		
**16a) CONSORT: For each group, number of participants (denominator) included in each analysis and whether the analysis was by original assigned groups**		
**16-i) Report multiple "denominators" and provide definitions**		
"The mean age of the participants was 28.9 (SD 9.1) years and 29.3 (SD 7.4) years for the video and AI groups, respectively. Both groups were predominantly female (n = 14, 88% for the video group; n = 12, 67% for the AI group). The education duration of the video group was 17.0 (IQR 16.0 to 17. 0) years, which was similar to the AI group with 16.0 (IQR 16.0 to 18.0) years. The two groups also had similar BMI results (21.0 [SD 2.9] kg/m2 for the video group; 21.3 [SD 2.4] hg/m2 for the AI group). "		
**16-ii) Primary analysis should be intent-to-treat**		
**17a) CONSORT: For each primary and secondary outcome, results for each group, and the estimated effect size and its precision (such as 95% confidence interval)**		
"Table 2 Primary and secondary outcomes change from baseline."		
**17a-i) Presentation of process outcomes such as metrics of use and intensity of use**		
**17b) CONSORT: For binary outcomes, presentation of both absolute and relative effect sizes is recommended**		
This study does not involve binary outcomes		
**18) CONSORT: Results of any other analyses performed, including subgroup analyses and adjusted analyses, distinguishing pre-specified from exploratory**		
This Results of any other analyses performed.		
**18-i) Subgroup analysis of comparing only users**		
**19) CONSORT: All important harms or unintended effects in each group**		
Nobody underwent any harm or unintended effect in each group of this study		
**19-i) Include privacy breaches, technical problems**		
**19-ii) Include qualitative feedback from participants or observations from staff/researchers**		
DISCUSSION		
**20) CONSORT: Trial limitations, addressing sources of potential bias, imprecision, multiplicity of analyses**		
**20-i) Typical limitations in ehealth trials**		
"Most of our participants had mild to moderate pain, whereas few had severe pain. The efficacy of AI-assisted exercise therapy for severe LBP is still unclear. Second, we only conducted 4 weeks of follow-up, with lasting efficacy. Sustained efficacy beyond this period remains to be explored. Finally, our participants were middle-aged or young. Whether our findings could be extrapolated to older adults is unknown."		
**21) CONSORT: Generalisability (external validity, applicability) of the trial findings**		
**21-i) Generalizability to other populations**		
**21-ii) Discuss if there were elements in the RCT that would be different in a routine application setting**		
**22) CONSORT: Interpretation consistent with results, balancing benefits and harms, and considering other relevant evidence**		
**22-i) Restate study questions and summarize the answers suggested by the data, starting with primary outcomes and process outcomes (use)**		
"This study examined the therapeutic effect of AI real-time guidance exercise therapy compared with conventional video guidance exercise therapy in young people with CNSLBP. We found that after 4 weeks of AI-assisted exercise therapy, the pain intensity, body function, psychology, and core muscle thickness had higher improvement compared to conventional exercise therapy."		
**22-ii) Highlight unanswered new questions, suggest future research**		
Our study had some limitations. First, most of our participants had mild to moderate pain, whereas few had severe pain. The efficacy of AI-assisted exerc ise therapy for severe LBP is still unclear. Second, we only conducted 4 weeks of follow-up, with lasting efficacy. Sustained efficacy beyond this period r emains to be explored. Finally, our participants were middle-aged or young. Whether our findings could be extrapolated to older adults is unknown.		
Other information		
**23) CONSORT: Registration number and name of trial registry**		
Clinical Trial Registry of China ChiCTR2300073185; https://www.chictr.org.cn/showproj.html?proj=198413		
**24) CONSORT: Where the full trial protocol can be accessed, if available**		
Clinical Trial Registry of China ChiCTR2300073185; https://www.chictr.org.cn/showproj.html?proj=198413		
**25) CONSORT: Sources of funding and other support (such as supply of drugs), role of funders**		
National Natural Science Foundation of China (Grant Nos. 82072528, 82002380), Natural Science Foundation of Guangdong Province (Grant No. 2022A1515012460), and National Health Commission multi-center collaborative horizontal research project (Grant No. DCMST-NHC-2019-AHT-01)		
**X26-i) Comment on ethics committee approval**		
**x26-ii) Outline informed consent procedures**		
**X26-iii) Safety and security procedures**		
**X27-i) State the relation of the study team towards the system being evaluated**		
